# Identification of optimum scopes of environmental drivers for schistosome-transmitting *Oncomelania hupensis* using agent-based model in Dongting Lake Region, China

**DOI:** 10.1017/S0031182024001306

**Published:** 2024-10

**Authors:** Junhui Huang, Jiamin Wang, Yanfeng Gong, Ning Xu, Yu Zhou, Liyun Zhu, Liang Shi, Yue Chen, Qingwu Jiang, Yibiao Zhou

**Affiliations:** 1 Fudan University School of Public Health, Building 8, Shanghai, China; 2 Key Laboratory of Public Health Safety, Fudan University, Ministry of Education, Building 8, Shanghai, China; 3 Fudan University Center for Tropical Disease Research, Building 8, Shanghai, China; 4 School of Epidemiology and Public Health, Faculty of Medicine, University of Ottawa, Ottawa, Canada

**Keywords:** agent-based modelling, environmental factors, *Oncomelania hupensis*, *Schistosomiasis japonica*

## Abstract

*Oncomelania hupensis* (*O. hupensis*), the sole intermediate host of *Schistosoma japonicum*, greatly influence the prevalence and distribution of schistosomiasis japonica. The distribution area of *O. hupensis* has remained extensive for numerous years. This study aimed to establish a valid agent-based model of snail density and further explore the environmental conditions suitable for snail breeding. A marshland with *O. hupensis* was selected as a study site in Dongting Lake Region, and snail surveys were monthly conducted from 2007 to 2016. Combined with the data from historical literature, an agent-based model of snail density was constructed in NetLogo 6.2.0 and validated with the collected survey data. BehaviorSpace was used to identify the optimal ranges of soil temperature, pH, soil water content, and vegetation coverage for snail growth, development and reproduction. An agent-based model of snail density was constructed and showed a strong agreement with the monthly average snail density from the field surveys. As soil temperature increased, the snail density initially rose before declining, reaching its peak at around 21°C. There were similar variation patterns for other environmental factors. The findings from the model suggested that the optimum ranges of soil temperature, pH, soil water content and vegetation coverage were 19°C to 23 °C, 6.4 to 7.6, 42% to 75%, and 70% to 93%, respectively. A valid agent-based model of snail density was constructed, providing more objective information about the optimum ranges of environmental factors for snail growth, development and reproduction.

## Graphical abstract

**Figure:**
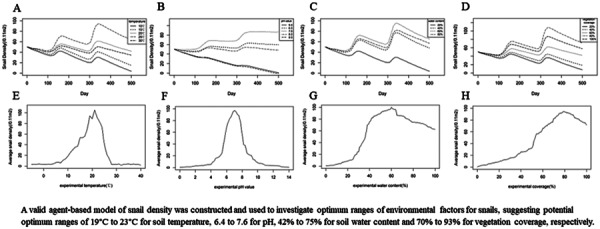

## Introduction

Schistosomiasis is caused by blood flukes (trematode) of the genus *Schistosoma*, with acute and chronic manifestations that significantly affect physical health and hinder social and economic development (Wang *et al*., 2022; Lv *et al.*, 2023; *Schistosomiasis*). There are approximately 12 000 deaths globally per year (Schistosomiasis). In China, schistosomiasis is caused by *Schistosoma japonicum* (*S. japonicum*) (Song *et al*., 2016; Gao *et al*., 2018; Li *et al*., 2021). Significant advances have been achieved in controlling schistosomiasis through more than 70 years of dedicated prevention measures (Song *et al*., 2016; Xia *et al*., 2017). However, it should be emphasized that the results of these efforts remain unstable and require further reinforcement for sustainable control (Li *et al*., 2020). Lake or marshland regions of Hunan, Hubei, Jiangxi, Anhui and Jiangsu Provinces with an appropriate environment for snail breeding, account for approximately 95% of current schistosomiasis cases, making it difficult to control the transmission of schistosomiasis (McManus *et al*., 2010). It is widely acknowledged that snail control and environmental management are important measures for schistosomiasis control (McManus *et al*., 2010; Schistosomiasis). *Oncomelania hupensis* (*O. hupensis*) serves as the sole intermediate host for *S. japonicum* in China (Xue *et al*., 2019; Shan *et al*., 2020), making snail control a crucial measure to prevent the transmission of schistosomiasis (Xu *et al*., 2021).

*O. hupensis* are amphibious freshwater molluscs whose breeding environment is influenced by various environmental factors, such as temperature, water level, soil conditions and vegetation conditions (Zhou *et al*., 2011; Wu *et al*., 2015). Furthermore, the distribution of *S. japonicum* is closely associated with that of *O. hupensis* (Xia *et al*., 2017), therefore, studying the ecological relationship between snail density and environmental factors contributes to schistosomiasis control (Gong *et al*., 2023). However, most current research on the relationship between *O. hupensis* and environmental factors remains limited to field investigations or laboratory studies (Nihei *et al*., 1998; Wu *et al*., 2014; Calata *et al*., 2019; Manalo *et al*., 2023), among which linear analysis methods constitute a considerably large portion (Qiu *et al*., 2014; Xia *et al*., 2017, 2020; Wang *et al*., 2022; Lv *et al.*, 2023). Furthermore, most studies rely on macroscopic descriptions without considering the complex system characteristics associated with snail growth, development, reproduction and distribution. It is recognized that the influence of environmental factors on snails is a complex systemic problem, therefore it is necessary to use more flexible and innovative methods to better understand the relationship between snails and environmental factors.

In recent years, scientific modelling methods for complex systems have received increasing attention in the field of public health (Tracy *et al.*, 2018), among which agent-based modelling has been widely used for epidemic control of common infectious diseases and has proven to be effective (Pizzitutti *et al*., 2015; Rutter *et al*., 2017). The agent-based model is a microscopic model that adopts a bottom–up approach to simulate the behaviour of agents and their interaction with the environment to evaluate their impact on the entire complex system (Tracy *et al.*, 2018). Agent-based modelling is characterized by autonomy, heterogeneity, feedback and randomness, making it more flexible in solving complex problems than ordinary linear mathematical models (Epstein, 2023). The agent-based model can simulate macrosystem behaviours by modelling the micro-behaviour of individual agents (Hu *et al.*, 2010). Similarly, taking *O. hupensis* as agents, an agent-based model might be used to simulate the overall dynamic change of snail density by modelling the micro-behaviour of snail agents. As an increasingly widely used tool for simulation, agent-based modelling has the ability to include more details about the mechanism of the system mechanism than traditional models (Hu *et al.*, 2010), providing an appropriate and feasible choice to model snail density. It can define the attributes of snail agents and environmental agents, track the actions of snail agents and their interactions with the environment, and yield outcomes at a population level (snail density) (Wang and Spear, 2015). Also, it can illustrate the dynamic changes in snail density through visualization. According to the characteristics of both snails and environment (Liu *et al.*, 2021), an agent-based model might be designed to simulate the interaction between snails and the environment. Therefore, this study aimed to establish a valid agent-based model of snail density and further analyse the optimum ranges of environmental factors for snail breeding, in order to provide a scientific basis for snail control.

## Materials and methods

### Study area

Dongting Lake, located in the northeast of Hunan Province and on the south bank of the Yangtze River, is one of the areas with the widest distribution of *O. hupensis* and the most severe schistosomiasis in China (Wu *et al*., 2014; Yang *et al*., 2018*b*). A bottomland of approximately 4.8 hm^2^ was selected as our study site in the East Dongting Lake area, which is a native ecological grassy marshland without artificial snail control (Yang *et al*., 2018*a*). This study area is classified as the eastern Asian monsoon climate zone, and the environment here is suitable and constitutes the habitat of *O. hupensis* snails (Li *et al*., 2007; Yang *et al*., 2018*a*). The flood season is generally from May to September every year (Yang *et al*., 2018*b*), and the period of marshland exposure period is generally from October to April of the following year (Li *et al*., 2016).

### Snail survey

The monthly *O. hupensis* snail surveys were carried out at the end of each non-flooded month (usually October through April of following year) from 2007 to 2016, and systematic quadrat sampling was adopted in the same site. Specifically, a 20 m × 20 m grid was established for the sampling points, resulting in a total of 120 quadrats using 8 lines, each containing 15 points. The survey lines, evenly spaced at 20 m intervals, were laid out on the ground in parallel. Along these lines, a 0.11 m^2^ iron wire frames were placed at every 20-m interval. The snails in each frame were collected and packed in bags marked with the corresponding lines and frame numbers. The snail density was then calculated as the average number of alive snails per frame (number/ 0.11 m^2^). The survey began on 30^th^, October 2007, and snail density was not investigated during the flood season.

### Statistical analysis

A snail survey database was created using Microsoft Excel 2019. Summary statistics included minimum and maximum snail density. The annual and monthly mean densities of *O. hupensis* from 2007 to 2016 were described. The calculations were performed in R 4.3.2.

### Model description and validation

#### Model construction and parameter setting

Using hierarchical modelling, the whole model system was divided into agent layer, environment layer, and agent-environment interaction layer.

The set of snail agents was constructed in the agent layer, whose properties and behaviour rules were established based on previous research results (Liang *et al.*, 2002; Jianqin *et al*., 2008; McCreesh *et al*., 2015). Each snail agent represents a snail, with growth status, lifetime, energy, and other attributes. The growth status included egg, young snail, mature snail and death. The average lifetime of the snails was 365 days. The growth energy represents the existing life-sustaining energy of the snail agent. According to a previous study (Hong *et al*., 2003), energy can be expressed as the accumulated temperature (d⋅°C), that is, the increase in daily temperature increase during snail development. When the accumulated temperature of the snail agent reaches a certain degree, it will reproduce.

The size of the environment layer corresponds to the method of system sampling, where 8 lines were set up, and each line had 15 points. Therefore, a total of 120 environment agents were set up. A snail survey frame represented an environmental agent, also known as a micro-environment for snail breeding, which was the living space and food source for snail growth, development and reproduction (Gong *et al.*, 2023). Each environmental agent had four environmental attributes based on previous studies (Jianqin *et al*., 2008), namely, soil temperature, soil pH, soil water content and vegetation coverage, which changed over time.

The interaction layer was used to describe the interaction between snail agents and environmental agents. Snail agents depended on the environment to survive, obtaining nutrition and energy from the environment, with vegetation serving as their primary source. At the same time, the behaviour of the snail agent was regulated by the environmental agent, with its growth, development, reproduction and distribution governed by certain rules, including movement rules (1), reproduction rules (2) and death rules (3). (1) Each snail agent in the model can move randomly in any direction. Snail eggs do not have the ability to move autonomously, and the movement rates of young and mature snails vary with temperature. Typically, movement is the fastest when the temperature ranges between 15  and 25°C (Jianqin *et al*., 2008). (2) The breeding behaviour of the snail agent must meet the following conditions: the snail agent is in the breeding period and has the minimum energy required for breeding, and the environmental conditions are suitable for snail breeding. The snails can reproduce when they reach maturity. The fecundity of mature snails varies under different temperature conditions, with the highest breeding speed occurring between 15 and 25°C (Jianqin *et al*., 2008). (3) The main cause of death among snails is the temperature and humidity of the environment. According to a previous study (Hu *et al.*, 2010), the mortality rate of snail was approximately 0.0054044/day.

The above model construction operations were performed in NetLogo 6.2.0. The variables and parameter ranges of the agent-based model (Table 1) were determined based on previous field investigations and laboratory studies (Hu *et al.*, 2010; Murphy *et al.*, 2016).

**Table 1.** Variables and parameter ranges of the agent-based model

Agent	variables	Variable instruction	Parameter range
Environment	Temperature	Soil temperature (°C)	−10 to 40
	pH	pH values of soil	0–14
	Water content	Soil water contention (%)	0–100
	Coverage	Vegetation coverage (%)	0–100
	Snail-num	The initial number of snails per survey frame	0–100
Snails	Lifetime	lifetime of each snail (days)	0–365
	State	The growth state of snails: 0 represents egg, 1 represents young snails, 2 represents mature snails and 3 represents dead snails	0, 1, 2, 3
	Energy	The energy of snails, also known as accumulated temperature, is the daily accumulation of heat accumulation (d⋅°C) of the snails in the microenvironment. When the heat accumulation reaches a certain value, it can reproduce	
	Min-energy	Minimum energy required for egg laying (d⋅°C)	4683
	Breeding-rate	Breeding rate of snails^a^	
	Mortality-rate	Snail mortality rate^a^	

a Indicates that the attributes change with environmental factors.

#### Output of the model result

After the agent-based model of snail density was constructed in NetLogo 6.2.0, the initial parameters of the model were assigned based on the results of our field survey in the marshland on 30 October 2009. The number of snails in each environmental agent in this model corresponded to the number of snails in the actual survey frame. The model was run according to the interactive rules of snails set by the model and the change rules of environmental factors. After running the model, the snail agents continued to move and interact with the environment according to the interactive rules from October to April in the following year. The results of the whole process were collected and further analysed in R 4.3.2.

#### Model validation

The validation of agent-based models includes two main stages, namely process validation and result validation (Murray *et al*., 2018). Model process validation mainly checks the consistency of the conceptual model of the agent-based model with the research purpose, given assumptions, existing theories and evidence (Murray *et al*., 2018). The data from field surveys were used to validate the agent-based model of snail density. The average snail density of marshland at the end of October from 2007 to 2016 was established as the initial snail density of the model and the modelled monthly snail density of the model was compared with the monthly average values of field surveys to verify the validity of the model output.

### Optimum ranges of environmental factors for snails

The agent-based model of snail density was run in NetLogo 6.2.0, and the trend of modelled snail density was observed. Then, BehaviorSpace, a tool provided by NetLogo 6.2.0, was used to perform simulation experiments to explore the optimal ranges of soil temperature, pH, soil water content, and vegetation coverage for snail growth, development and reproduction. When the optimum range of environmental factors for snails was explored, the initial density of snails was set to 50 snails/0.11 m^2^. The optimal soil temperature, pH value, soil water content and vegetation coverage for snail breeding were explored from −10 to 40°C, 0–14, 0–100% and 0–100%, respectively. After adjusting various environmental parameters, the changing trend of snail density was obtained after 500 steps of model operation under different suitable environmental conditions. Each experiment simulated in BehaviorSpace was conducted 50 times to reduce random errors and find the appropriate range of environmental factors. The influence of different environmental factors on the dynamic change in snail density was then compared to determine the optimal ranges that maintained the population size relatively stable or demonstrated the fastest increase, representing the appropriate range of environmental factors. All results were visualized in R 4.3.2.

## Results

### Snail density

**Figure 1.**
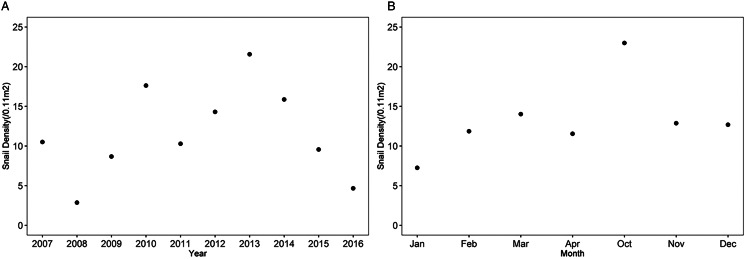
Average density of snails in the bottomland from 2007 to 2016. (A) annual average density of snails, (B) monthly average density of snails.

The yearly average density of *O. hupensis* in the marshland of the Dongting Lake Region fluctuated between 2007 and 2016 (Fig. 1A). It was the highest in 2013 (21.563 snails/0.11 m^2^) and the lowest in 2008 (2.861 snails/0.11 m^2^). The monthly average snail density was highest in October (22.985 snails/0.11 m^2^) and the lowest in January (7.251 snails/0.11 m^2^) (Fig. 1B).

### Model construction and validation

**Figure 2.**
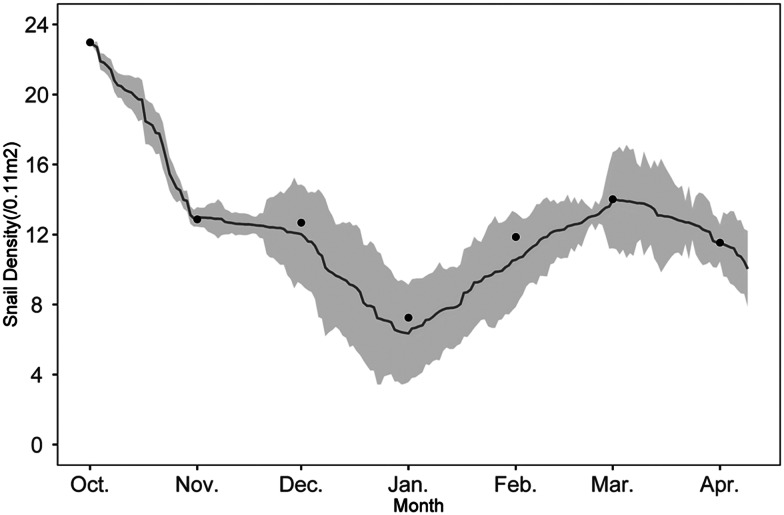
Validation of agent-based model of snail density. The grey line represents the average changing trend of modelled snail density, while the grey shadow represents the 95% confidence intervals for snail density. The dots represent the monthly average observed snail density in the marshland from 2007 to 2016.

The model was constructed in NetLogo6.2.0 and validated using the monthly average density of snails from 2007 to 2016. The average snail density in October from 2007 to 2016 was 22.985 snails/0.11 m^2^, which was set as the initial snail density of the agent-based model. The model was run to export the dynamic changes in snail density from October to April of the following year, and a 95% confidence interval was calculated. The modelled snail density showed good agreement with the monthly average snail density of the field surveys, with no statistical difference between the two models. As shown in Fig. 2, the changing trend of the modelled snail density was consistent with the monthly average snail density in field surveys, with the highest modelled snail density in October (22.834 snails/0.11 m^2^) and the lowest one in January (6.348 snails/0.11 m^2^). The 95% confidence intervals basically encompassed the monthly average snail density from field surveys (which was represented by the dots in Fig. 2), signifying the model's reliability. Furthermore, the 95% confidence interval for the density of the modelled snails was the narrowest [(12.929 ± 0.474) snails/0.11 m^2^] in November, gradually widening until January [(7.231 ± 3.794) snails/0.11 m^2^]. Subsequently, the confidence interval of snail density showed a narrowing trend.

### Optimum ranges of environmental factors for snails

#### Optimal range of soil temperature for snails

**Figure 3.**
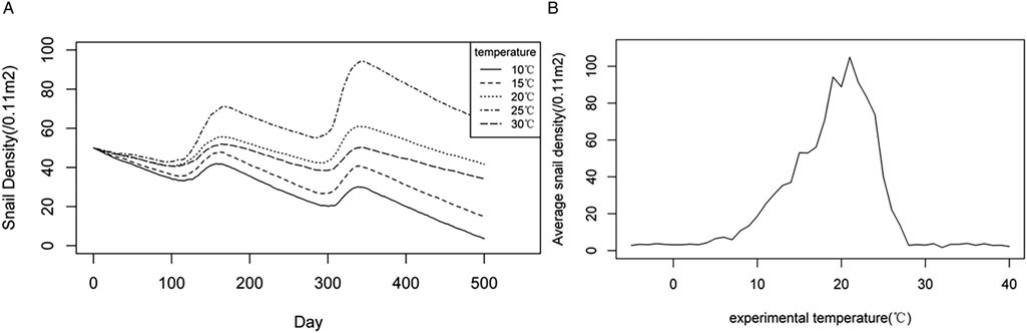
Changing trends of snail densities under different experimental soil temperatures: (A) dynamic changes of snail densities with time, (B) average snail densities over a 5-year period under different temperatures.

Using the agent-based model to create experiments in BehaviorSpace, the optimal soil temperature for the growth, development and reproduction of *O. hupensis* was assumed to be 10°C, 15°C, 20°C 25°C and 30°C, respectively. Each experimental temperature was repeated 50 times, totalling 250 experiments. As shown in Fig. 3A, the average densities of snails at different optimal temperatures were tracked over time. All experimental results showed similar seasonal trends as time passed. Around 100-day mark of model operation, corresponding to February of the following year in reality, a decreasing trend in snail density was observed. Subsequently, the density of snails experienced a slight increase, followed by another decrease around the 140-day mark. As the model approached about 300 days, corresponding to August of the following year in reality, the snail density dropped to a minimum, before increasing once again. When the experimental soil temperature was set at 20 °C, the snail density showed an overall increasing trend. However, at an experimental soil temperature of 15°C or 25°C, the overall density of the snails was relatively stable. On the contrary, at experimental temperatures of 10°C and 30°C, the snail density showed a significant decreasing trend.

Further experiments were created for each soil temperature from −10°C to 40°C. Figure 3B shows that each experiment was carried out for 5 years and repeated 5 times, with the average snail density corresponding to each temperature recorded and plotted. Figure 3B demonstrated a non-linear relationship between snail density and soil temperature. The average density of snails remained low until an experimental temperature of 8°C. When the experimental temperature increased from 8°C to 20°C, the snail density experienced a rapid increase, reaching its peak at 20°C, and was followed by a sharp decline. When the experimental temperature was set at 28°C or higher, the snail tended to approach 0 snails/0.11 m^2^. When the experimental temperature ranged between 15°C and 24°C, the average snail densities over a 5-year period exceeded the initial snail density of 50 snails/0.11 m^2^. When the experimental temperature was set between 19°C and 23°C, the average snail density remained constantly high level, surpassing 80 snails /0.11 m^2^. The results indicated that 19–23°C could be the optimal soil temperature range for snail growth, development and reproduction.

#### Optimal range of pH value for snails

**Figure 4.**
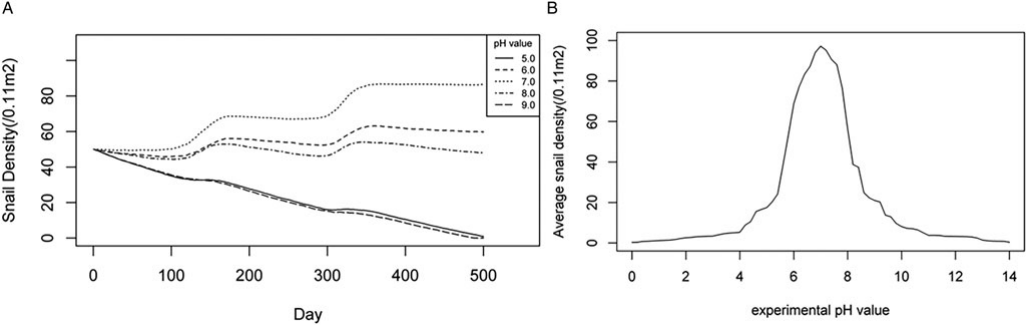
Changing trends of snail densities under different experimental pH values: (A) dynamic changes of snail densities with time, (B) average snail densities over a 5-year period under different pH values.

The optimal soil pH for snails was assumed to be 5.0, 6.0, 7.0, 8.0 and 9.0, respectively. Each experimental pH value was repeated 50 times, totalling 250 experiments. As shown in Fig. 4A, the average densities of *O. hupensis* under different optimal pH values were tracked over time and a similar seasonal trend was observed as time progressed. However, when the experimental pH was set at 7.0, there was an evident increasing trend in snail density. At an experimental pH of 6.0 or 8.0, the snail density was relatively stable overall, while at an experimental pH of 5.0 or 9.0, the overall snail density showed a significant downward trend. In particular, when the experimental pH value was set to 9.0, the snail density dropped to 0 after close to 500 days.

To further explore the optimal range of soil pH values for snail breeding, experiments were conducted for each soil pH value from 5.0 to 9.0 at an interval of 0.2 for 5 years. Each experiment was repeated 5 times, and the average snail density at each pH was recorded and plotted. As shown in Fig. 4B, there was a non-linear relationship between snail density and soil pH value. The snail density remained low when the experimental pH value was below 4.0, increased with pH until 7.0 and then decreased. When the experimental pH value exceeded 10.6, the density of snails tended towards 0. According to the dynamic changes in snail density under different experimental pH values, when the experimental pH value was between 5.8 and 8.0, the average snail densities over a 5-year period were higher than the initial snail density (50 snails/0.11 m^2^). Furthermore, when the experimental pH value was set from 6.4 to 7.6, the average density of snails remained constantly high, exceeding 80 snails /0.11 m^2^. The findings suggested that the appropriate range of soil pH for snails might be 6.4–7.6.

#### Optimal range of soil water content for snails

**Figure 5.**
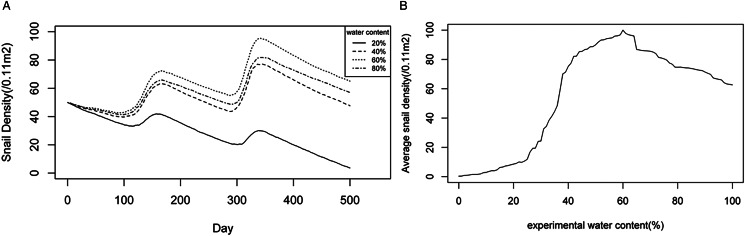
Changing trends of snail densities under different experimental soil water contents: (A) dynamic changes of snail densities with time, (B) average snail densities over a 5-year period under different soil water contents.

The optimal soil water content for snails was assumed to be 20%, 40%, 60% and 80%, respectively, in the agent-based model. Each experimental water content was conducted 50 times, totalling 200 experiments. As shown in Fig. 5A, the average densities of *O. hupensis* under different experimental soil water contents were tracked over time. There were similar seasonal trends as time progressed. When the water content was set to 40%, 60% or 80%, the snail density showed obvious upward trends, with the highest increase observed at 60% water content. On the contrary, when the soil water content was set to 20%, there was a noticeable decrease in snail density.

To further explore the optimal range of soil water content for snail breeding, experiments were conducted for each optimal soil water content between 0 and 100% for 5 years, each experiment repeated 5 times. Figure 5B illustrated a non-linear relationship between snail density and soil water content. The snail density remained low when the experimental water content was below 28%, increased rapidly with the water content until it reached 40%, and then increased gradually. However, when the experimental water content exceeded 60%, the snail density steadily decreased. When the experimental soil water content exceeded 37%, the average snail densities over a 5-year period were higher than the initial snail density of 50 snails/0.11 m^2^. Furthermore, when the experimental water content was established between 42% and 75%, the average snail density remained consistently high, exceeding 80 snails /0.11 m^2^. These findings suggested that the optimum range of soil water content for snails could be 42–75%.

#### Optimal range of vegetation coverage for snails

**Figure 6.**
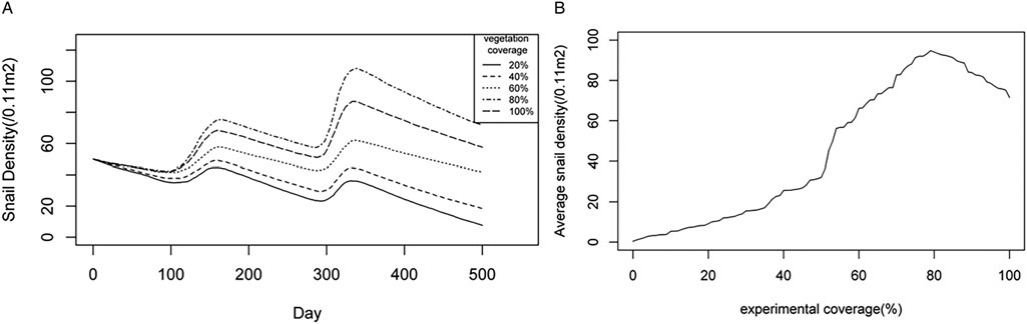
Changing trends of snail densities under different experimental vegetation coverage levels: (A) dynamic changes of snail densities with time, (B) average snail densities over a 5-year period under different vegetation coverage levels.

The optimal vegetation coverage suitable for snails was assumed to be 20%, 40%, 60%, 80% and 100%, respectively, in the agent-based model. Each experimental vegetation coverage was conducted 50 times, totalling 250 experiments. As shown in Fig. 6A, there were similar seasonal trends as time progressed, which were in accordance with the ecological laws of snails. However, when experimental vegetation coverage exceeded 60%, there were significant increases in snail density. The snail density was the highest, with vegetation coverage being 80%. The snail density was relatively stable when the vegetation coverage was 60%. However, when vegetation coverage was below 40%, there were significant declines in snail density.

To further explore the optimal range of vegetation coverage for snail breeding, experiments were conducted for each optimal vegetation coverage between 0 and 100% for 5 years. Each experiment was repeated 5 times, and the average snail density was recorded and plotted for each vegetation coverage. Figure 6B illustrates a non-linear relationship between snail density and vegetation coverage. The snail density increased slowly when the experimental vegetation coverage was below 50%, and then rapidly increased to 79%, followed by a slight decrease afterwards. When the experimental vegetation coverage exceeded 54%, the average snail densities over a 5-year period were higher than the initial snail density of 50 snails/0.11 m^2^. Furthermore, when the experimental vegetation coverage was established between 70% and 93%, the average snail density remained consistently high, exceeding 80 snails/0.11 m^2^. The proper vegetation coverage for snails might be in the range of 70–93%.

## Discussion

Agent-based models are bottom–up models (Tracy *et al.*, 2018), and a key challenge in model construction is to ensure that the results correctly reflect the real-world impacts (Murray *et al*., 2018). The choice of model structure has a great impact on the reproduction ability of the real system and the randomness of the output results, posing great challenges to model validation (Yang and Ze-jian, 2013; Rutter *et al*., 2017; Russo *et al*., 2022). The combination of process validation and result validation can effectively enhance the validity of the model (Jianqin *et al*., 2008; Pizzitutti *et al*., 2015). We constructed the model by including the main attributes of snail agents, environmental agents, and complex interaction rules between them, and found that the dynamic changing trend of the modelled snail density was consistent with the monthly average snail density from field surveys.

This model showed a seasonal trend closely similar to that in real-world conditions. Over the course of 100 days, corresponding to February of the following year in reality, the temperature gradually rose in early spring, prompting an increase in snail density. As environmental conditions became favourable and energy levels accumulated for spawning, mature snails began to lay eggs and eventually died after reaching their maximum lifespan. By the 200th day, corresponding to May of the following year, snail eggs hatched to young snails, marking the onset of a new life cycle. Although summer flooding led to a temporary decrease in snail density, it also created an ideal environment for egg hatching, thereby facilitating an overall growth in snail density. Under the environmental conditions set by the model and the behaviour rules of snails, the dynamic changes in snail density closely mirrored the trend of natural reality. Therefore, the modelled snail density presented strong agreement with the monthly average snail density observed in the field surveys, suggesting that the model has been quantitatively validated to a certain extent and demonstrates good reliability. These findings indicate the feasibility of the agent-based model to predict changes in snail density within a certain time frame.

The geographic distribution of *O. hupensis* has been reported to be restricted to area south of the 33°15′N latitude (Yang *et al*., 2007), and soil temperature is known to play an important role in the growth and development of *O. hupensis* (Xu *et al*., 2023). According to the agent-based snail density model in this study, it was found that soil temperatures ranging from 15°C to 24°C was suitable for snail breeding, with temperatures between 19°C and 23°C specifically promoting an increase in snail density. Soil temperature exceeding 28°C or dropping below 8°C might be not conducive to the survival of snails. Previous literature revealed that soil temperatures ranging from 15°C to 20°C was suitable for snail mating, and extreme temperatures outside could disrupt or inhibit their mating activity. In addition, the temperature range for egg laying is generally between 20°C and 25°C, providing optimal conditions for egg development and survival (Manalo *et al*., 2023). Laboratory research has also suggested that the optimal range of soil temperature for *O. hupensis* is between 20°C and 30°C, with temperature below or above this range potentially causing delayed or interrupted development and reproduction (Yang *et al*., 2007). A previous research by Wu *et al*. (Wu *et al*., 2014), reported the suitable range of soil temperature for snail breeding was 22.73–24.23°C. Furthermore, a meta-analysis conducted by Liu *et al*. (Liu *et al.*, 2021) suggested that the suitable range of soil temperature for snail breeding was 16–20°C. The model-derived optimal soil temperature range aligns fundamentally with the outcomes of both laboratory experiments and field surveys, notwithstanding the varying scope of these ranges (Wu *et al*., 2014; Liu *et al.*, 2021). This concordance may be ascribed to the selection of study sites and the duration of the study periods, which could have influenced the results. In light of these findings, we suggest that the optimal soil temperature for snails is between 19°C and 23 °C.

The soil pH value is also one of the important environmental factors affecting snail breeding. Extreme pH values, either too high or too low, can adversely affect the shell of snails and subsequently impede snail growth. The pH of the soil also affects the solubility of soil minerals and the absorption of nutrients, indirectly influencing snail density (Manalo *et al*., 2023). The study determined that a pH range of 5.8 to 8.0 is conducive to snail breeding, with the narrower band of 6.4 to 7.6 being particularly effective in enhancing snail density. Similarly, a study using the geographical weighted regression method (Wu *et al*., 2014), reported that soil pH ranging from 6.6 to 7.0 might be suitable for snail breeding. These outcomes are in harmony with a meta-analysis conducted by Liu *et al*. (Liu *et al.*, 2021) who indicated that a soil pH value between 5.5 and 7.0 is appropriate for snail propagation. Our results not only support but also reinforce these conclusions. Synthesizing the cumulative evidences from prior researches and this study, a pH range of 6.4 to 7.6 is the most conducive environment for snail breeding.

*O.hupensis* are amphibious, and water is the most important ecological factor for snail reproduction, growth, development and diffusion (Zhao *et al.*, 2014). Findings from the agent-based model indicated that a water content of 37% or greater, especially between 42% and 75%, was suitable for snail breeding. Wu *et al*. (Wu *et al*., 2014) reported a possible optimum scope of water content ranging from 58.70% to 68.93%. However, other studies proposed that a range of 59% to 80% or a range of 53% to 70% is suitable water content for snail breeding (Zhao *et al.*, 2014; Liu *et al.*, 2021). The discrepancies observed in these results are primarily ascribed to different study sites and application of distinct statistical methodologies. Integrating the findings from existing literatures with the outcomes of this study, we propose that a range of 42% to 75% could be deemed as the most favourable water content for snail breeding.

Vegetation conditions are crucial for snail breeding. They can provide food for snails and shade them from the sun. However, *Oncomelania* snails also need adequate light to survive, and as a result, too little or too much vegetation is not conducive to snail growth (Zhao *et al.*, 2014). In generalized additive models for the relationship between *O. hupensis* density and micro-environmental factors, vegetation coverage demonstrated a *U*-shaped or more complex relationship with snail density (Liu *et al.*, 2021). This research revealed that a vegetation coverage of 54% or higher was suitable for snails to grow, develop and reproduce, with a range of 70–93% promoting increased snail density. Similarly, Liu *et al*. (Liu *et al.*, 2021) demonstrated that a vegetation coverage range of 60–100% was a suitable range for snail breeding. Our results were basically consistent with results from field surveys and laboratories (Zhao *et al.*, 2014; Liu *et al.*, 2021), and the minor discrepancies observed among the studies might arise from a multitude of factors, including but not limited to methodological approaches, sample selection, and experimental conditions. Combined with the results of this study and other relevant research evidence from prior fieldwork and laboratories, it is suggested that a vegetation coverage ranging between 70% and 93% could be considered as the most favourable habitat for snails.

Previous studies examining the relationships between environmental factors and snails relied mainly on laboratory studies or field investigations, each with inherent limitations. Laboratory studies often focused on isolating the influence of specific environmental factors on snails, but may overlook the intricate interactions among these factors. On the contrary, field investigations lack controlled conditions, making it challenging to attribute observed effects solely to environmental variables. The agent-based model in this study not only controlled the environmental factors, but also incorporated the dynamic interaction between the environment and snails. This approach offers a novel perspective for examining the relationship between snail density and environmental factors. In contrast to previous studies, this study conducted simulated experiments in BehaviorSpace, allowing for a broader exploration of the subject matter. Furthermore, the optimal environmental conditions for snail breeding obtained in this study are basically consistent with the results of previous laboratory or field investigations. Although the studies reveal minor discrepancies that could stem from various factors like methodological approaches, sample selection and experimental conditions, these variations do not undermine the overall robustness and validity of our findings, but add depth and complexity to our understanding of the relationship between environmental factors and snails. The presence of these variations also serves as a reminder of the dynamic and complex nature of the snail agents, which cannot be fully captured by a single study. These encourage a more cautious approach and promote a deeper exploration of the underlying mechanisms between environmental factors and snail density. The outcomes of this model not only reinforce the validity of agent-based model construction but also enforce our understanding of the relationship between snail density and environmental factors.

However, our study has several limitations. Firstly, while we accounted for several key properties of snails and environment in our simulations, there may exist more complex interactions that were not fully considered. Besides, there might also be other organisms that could compete with *O. hupensis* for resources in the environment and other pathogens that could affect snail density. Second, only 4 main environmental factors were included in our agent-based model, not considering types of vegetation, rainfall and water level. However, it should be noted that the soil water content, included in our model, partially reflects rainfall and water level to some extent.

## Conclusion

This research constructed a robust agent-based model of snail density and investigated optimum ranges of environmental factors for snail growth, development and reproduction. The analysis revealed an inverted *U*-shaped relationship between environmental factors and snail density, suggesting potential optimum ranges of 19°C to 23°C for soil temperature, 6.4 to 7.6 for pH, 42% to 75% for soil water content and 70% to 93% for vegetation coverage, respectively. These findings contribute to a better understanding of the relationship between snail density and environmental factors, thus aiding in the development of effective snail control strategies.

## Data Availability

Data available on request from the authors.
